# Demand preferences for health management services in a population of older adults with visual impairment in China: a conjoint analysis

**DOI:** 10.1186/s12877-022-02957-0

**Published:** 2022-03-26

**Authors:** Shuqin Li, Ai-ping Wang

**Affiliations:** The First Affiliated Hospital of China Medical University, Shenyang, Liaoning Province China

**Keywords:** Older adults, Visual impairment, Health management, Service, Demand, Preferences

## Abstract

**Background:**

Visual impairment (VI) seriously affects the quality of life of the older adults. It is important to formulate appropriate health management strategies for the older adults with VI to help delay the disease development and progression, and improve life quality. The aim of this study was to understand the demand preference for health management services of the older adults with VI, and to provide a reference for the development of future health management strategies in this population.

**Methods:**

The conjoint analysis method was used to analyze demand preferences for health management services of the older adults with VI. 11 keywords were extracted after literature analyzed, 6 keywords were selected as the attributes of health management strategy after expert discussion and the level of each attribute was determined. Then 18 representative virtual health management strategies were formed by combination of different attribute levels through orthogonal design, and older adults with VI were asked to score. A total of 334 older adults with VI who attended the ophthalmology department of the First Affiliated Hospital of China Medical University and the Fourth People's Hospital of Shenyang from February 27, 2021 to June 30, 2021were enrolled in this study by stratified sampling. Of the 334 included people, 80 had grade 1 VI, 80 had grade 2 VI, 84 had grade 3 VI, and 90 had grade 4 VI.

**Results:**

The relative importance of health management services ranked by older adults with VI was continuing care (24.033%), visual aid application (19.61%), health education (16.241%), preventive healthcare (15.667%), safety management (12.757%), and rehabilitation training (11.392%). The utility values of each level of continuing care, safety management and preventive healthcare were positive, whereas the utility values of each level of visual aid application, health education and rehabilitation training were negative. The relative importance and utility values of health management services were different for the older adults with different grades of VI.

**Conclusions:**

From the whole group, the older adults with VI have a higher preference for continuing care and a lower preference for rehabilitation training. The preference of the older adults with different grades of VI is different, so medical workers can formulate corresponding health management strategies according to their different demand preferences, and carry out hierarchical health management. Services that they preferred should be satisfied as much as possible in the health management strategy, while the reasons for the services with lower preference can be explored and make targeted improvement to meet the demand preferences of them.

## Background

The global population is aging rapidly. Between 2015 and 2050, it is estimated that the proportion of the global population aged 60 or older will almost double, from 12 to 22% [1]. In 2020, in China, the population aged 60 and older exceeded 264 million, accounting for 18.7% of the total population. It is expected that by 2050, in China, the number older adults aged 60 and older will be close to 500 million, accounting for more than one third of the total population.

Visual impairment is a common condition among the older adults, includes blindness and low vision, and is defined as reduced visual acuity and field of view in both eyes due to various causes that cannot be corrected, affecting daily life and social participation [2]. The prevalence of VI increases with age [3]. It is projected that the number of people worldwide with moderate and severe VI will increase from 217 million in 2015 to 588 million in 2050, with 70% of those being 50 and older [4]. VI has become an important public health problem which not only increases economic burden, but also reduces the quality of life and increases the risk of death [5]. In addition, people with VI reported greater barriers to health care access and limited access to health promotion information [6].

Older adults with VI lose part or all of their visual ability, which reduces their ability to live independently and social participation, often resulting in withdrawal from all social role [7]. In addition, VI is also a risk factor for other age-related diseases, as older adults with VI are twice as likely to have falls and four to eight times as likely to experience hip fracture than older adults without VI. Depression incidence is three times higher and the average time for admission to the nursing home is three years earlier in older adults with VI than without [8, 9].VI is a serious condition which affects the physical and mental health of the older adults, leading to poor quality of life and greater dependence on others [10]. The main causes of VI include cataract, glaucoma, age-related macular degeneration, diabetic retinopathy, and uncorrected refractive errors, among which 80% can be prevented [11]. Therefore, it is necessary to provide timely targeted health management services for the older adults, to help delay the development of visual impairment and improve life quality.

There have been many studies on the application of visual aids and rehabilitation services for older adults with VI [12–15]. In recent years, there is a growing awareness of the importance of preventive healthcare and studies on visual screening, lifestyle intervention gradually increased. These services have been proved to be effective in delaying the deterioration of vision [16, 17]. Low vision rehabilitation centers in Germany, Australia and some countries are well developed, can provide comprehensive health management services for patients with VI [18, 19]. However, there are few rehabilitation centers for low vision in China, and the health management of patients with VI is mostly managed by hospitals. Two-way management between hospitals and communities as well as family intervention has gradually emerged, which provides a lot of convenience for patients with VI [20, 21]. However, patients' compliance is different for each service, which is worthy of further study [22, 23].

The world report on vision, recently released by WHO [24], proposes an Integrated People-Centered Eye Care (IPEC), emphasizing provision of accessible eye care that is sensitive to local needs. How to provide effective services for the older adults with VI that target physical and mental health, self-care ability and social function has been the focus of attention of our society [25, 26]. In recent years, studies on the needs of patients with VI have focused on specific vulnerable groups, such as people with low-income and refugees, as well as the older adults. However, most studies have mainly focused on existent needs rather than analyzing specific service demands [27–30]. In this study, we analyzed the specific service demand preferences of the older adults with different grades VI, provide a reference for the formulation of targeted health management strategy to help delay the deterioration of VI in the older adults, improve their quality of life and promote healthy aging.

## Materials and methods

### Respondents

According to the Standardization Administration of China [2], VI is classified into four grades. Grade1: lightless sensation, or the best corrected visual acuity (BCVA) < 0.02, or visual field radius < 5°; Grade2: the BCVA is 0.02 ~  < 0.05, or visual field radius < 10°; grade3: the BCVA is 0.05 ~  < 0.1; Grade4: the BCVA is 0.1 ~  < 0.3. Based on the criteria, we conducted stratified sampling among the older adults with VI. We divided the subjects into four layers, corresponding to four grades of VI respectively, and enrolled the subjects continuously from February 27, 2021 until the sample size requirements of each layer were met. The inclusion criteria of respondent were: age of 60 and older; the VI standards set by Standardization Administration of China were met; voluntary participation and signed informed consent. The exclusion criteria were: diagnosis with mental illness or dementia and unable to communicate verbally; all critical diseases that precluded cooperation with the investigator.

Based on previous literature, the minimum sample size for the conjoint analysis method is 75 [31]. A total of 334 older adults with VI who were continuously enrolled in the ophthalmology outpatient department and ward of the First Affiliated Hospital of China Medical University and the Fourth People's Hospital of Shenyang from February 27, 2021 to June 30, 2021, were included in this study. Of the 334 included people, 80 had grade 1 VI, 80 had grade 2 VI, 84 had grade 3 VI, and 90 had grade 4 VI. This study was approved by the Ethics Committee of the First Affiliated Hospital of China Medical University.

### Methods

In this study, we used conjoint analysis to analyze the demand preference for health management services in a population of older adults with VI. We have first carried out a literature analysis method and sought expert advice to determine the attributes and levels of health management strategy. Then, we obtained typical virtual health management strategies through orthogonal design, and cards containing a description of the strategy were made for each virtual strategy. Finally, a population of older adults with VI was asked to score the virtual health management strategies formed by the level combination of different attributes based on their demand preferences [32].

#### Determination of the attributes and levels of health management strategy

**Fig. 1:**
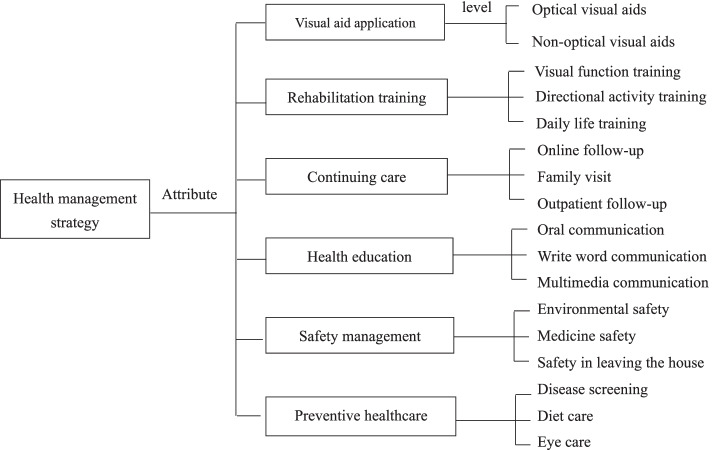
Attributes and levels of health management strategy

Based on relevant literature, health management services were extracted as keywords, and proven effective services were summarized and analyzed. The retrieval databases used include PubMed, Embase, CINAHL, CNKI, Wanfang and CBM from January 1, 2000 to January 20, 2021. Relevant studies were selected using a combination of keywords including((“elderly”[Mesh]) OR (“aged”[Mesh]) OR elderly[Title/Abstract] OR aged[Title/Abstract] OR old*[Title/Abstract]) AND ((“Visually Impaired Persons”[Mesh]) OR visual* impairment[Title/Abstract] OR (“Vision, Low”[Mesh]) OR low vision[Title/Abstract]). A total of 11 keywords were extracted, including visual aid application, rehabilitation training, continuing care, health education, vision screening, safety management, psychological nursing, online health management, social support, lifestyle intervention and nursing of traditional Chinese medicine. psychological nursing, social support and nursing of traditional Chinese medicine were deleted after expert discussion, vision screening and lifestyle intervention were summarized as preventive healthcare, Finally, 6 keywords were selected as the attributes of health management strategy. The specific information of experts is shown in Table 1, the attributes and levels of health management strategy were finally determined as shown in Fig. 1.

**Table 1:** Specific information of 7 invited experts

NO	Education degree	Title	Major	Years of working
Z1	Doctor	Professor	Ophthalmology	19
Z2	Doctor	Professor	Ophthalmology	22
Z3	Doctor	Professor	Ophthalmology	19
Z4	Doctor	Professor	Ophthalmology	35
Z5	Master	Professor	Nursing	28
Z6	Doctor	Associate Professor	Ophthalmology	20
Z7	Doctor	Associate Professor	Ophthalmology	17

#### Virtual health management strategy simulation and orthogonal design

According to the attributes and levels obtained in the previous, health management service at different levels were arranged and combined to obtain different virtual health management strategies. To avoid too many combinations, which would make the evaluation work tedious, we adopted an orthogonal design to create a set of representative virtual health management strategies with different attribute level combinations. A total of 18 typical combinations were optimized (Table 2), and cards are made for each one of them to appropriately describe the strategy.

**Table 2:** 18 typical health management strategies

NO	Visual aid application	Rehabilitation training	Continuing care	Health education	Safety management	Preventive healthcare
1	Non-optical	Directional activity training	Family visit	Write word	Safety in leaving the house	Disease screening
2	Optical	Directional activity training	Family visit	Multimedia	Environmental safety	Disease screening
3	Non-optical	Daily life training	Online follow-up	Multimedia	Safety in leaving the house	Diet care
4	Non-optical	Daily life training	Family visit	Oral	Environmental safety	Diet care
5	Non-optical	Directional activity training	Outpatient follow-up	Oral	Medicine safety	Eye care
6	Optical	Directional activity training	Outpatient follow-up	Write word	Environmental safety	Diet care
7	Optical	Daily life training	Outpatient follow-up	Multimedia	Medicine safety	Disease screening
8	Optical	Directional activity training	Online follow-up	Oral	Safety in leaving the house	Eye care
9	Non-optical	Visual function training	Outpatient follow-up	Multimedia	Environmental safety	Eye care
10	Optical	Directional activity training	Online follow-up	Multimedia	Medicine safety	Diet care
11	Optical	Visual function training	Family visit	Multimedia	Safety in leaving the house	Eye care
12	Optical	Visual function training	Family visit	Oral	Medicine safety	Diet care
13	Non-optical	Visual function training	Online follow-up	Write word	Medicine safety	Disease screening
14	Optical	Daily life training	Outpatient follow-up	Oral	Safety in leaving the house	Disease screening
15	Optical	Visual function training	Outpatient follow-up	Write word	Safety in leaving the house	Diet care
16	Optical	Daily life training	Family visit	Write word	Medicine safety	Eye care
17	Optical	Visual function training	Online follow-up	Oral	Environmental safety	Disease screening
18	Optical	Daily life training	Online follow-up	Write word	Environmental safety	Eye care

#### Data collection

We designed a general information questionnaire and cards describing 18 virtual health management strategies as mentioned above. The questionnaire included information about age, sex, education level, current residence, medical insurance status, eye disease diagnosis, duration of illness, other presence of chronic diseases. Older adults with VI were asked to score their preferences of health management strategies according to a Likert scale of 9, where 1 meant a “I would not want it at all” response and 9 meant a “I would want it very much” response. The research was conducted face-to-face. Researchers will explain the purpose and significance of this study in detail before the beginning of the survey, the specific meaning of each health management service will also be explained in detail, and examples will be given to ensure that participants fully understand before scoring.

#### Calculation of the utility value

We collected the preference values for each attribute of health management strategy in a population of older adults with VI. We assessed the relative importance of each attribute in the context of the overall strategy. The positive and negative of utility value reflects the attitude of older adults with VI to each level, and the utility value reflects the degree of liking or disliking of a certain attribute.

### Statistical analysis

All data were analyzed with SPSS v26.0. Frequency and percentages were used for descriptive statistics of basis information, comparison between groups was performed using χ^2^test, and α = 0.05 was set as the test level. The study adopts the full-profile analysis method of the conjoint analysis, which traditionally use the Ordinary least square (OLS) model to estimate parameters. Each independent variable represents the presence or absence of an attribute or level, and the dependent variable is the evaluation score of the respondents on a health management strategy. Entering the results of Likert scale into SPSS26.0 and running the conjoint analysis program automatically generates relative importance and utility values. The analysis of variance was used for group comparison, with α = 0.05 set as the test level.

## Results

### General information

A total of 334 questionnaires were sent out and were all received (effective recovery of 100%). There was no significant difference in age, sex, education level, current residence, medical insurance status, eye disease diagnosis, duration of illness, other chronic conditions among older adults with VI of different grades (*P* > 0.05). Of the 334 respondents 151 were male (45%) and 183 were female (55%). The majority were aged between 60 and 70 years, accounting for 60% of the population enrolled. Half of respondents had junior high school education and the majority lived in cities (78%). The most common diagnosis was cataract (70%), followed by diabetic retinopathy (20%), glaucoma (16%) and age-related macular degeneration (11%), with 41.6% had two or more eye diseases at the same time. 90% of the patients had a disease course of less than 5 years, and 64% of the respondents also had other chronic diseases, mostly diabetes (44%) and hypertension (38%) (Table 3).

**Table 3:** Baseline characteristics of the respondents

	Grade 1 (*n* = 80)	Grade 2 (*n* = 80)	Grade 3 (*n* = 84)	Grade 4 (*n* = 90)	Total (*n* = 334)	*P*
**Sex**						
Male	36(45%)	37((46%)	39(46%)	39(43%)	151(45%)	0.975
Female	44(55%)	43(54%)	45(54%)	51(57%)	183(55%)	
**Age**						
60 ~ 70	45(56%)	51(64%)	47(56%)	57(63%)	200(60%)	0.83
71 ~ 80	26(33%)	21(26%)	30(36%)	26(29%)	103(31%)	
81 ~ 90	9(11%)	8(10%)	7(8%)	7(8%)	31(9%)	
**Education Level**						
Primary and below	20(25%)	20(25%)	20(24%)	23(26%)	83(25%)	0.659
Junior high school	39(49%)	36(45%)	44(52%)	51(57%)	170(51%)	
Senior high school	8(10%)	18(23%)	11(13%)	10(11%)	47(14%)	
University and above	13(16%)	6(7%)	9(11%)	6(7%)	34(10%)	
**Current residence**						
City	60(75%)	64(80%)	67(80%)	70(78%)	261(78%)	0.859
Country	20(25%)	16(20%)	17(20%)	20(22%)	73(22%)	
**Medical Insurance**						
with	76(95%)	78(97%)	80(95%)	85(94%)	319(96%)	0.792
without	4(5%)	2(3%)	4(5%)	5(6%)	15(4%)	
**Diagnosis**						
Cataract	52(65%)	54(68%)	56(67%)	73(81%)	235(70%)	0.23
Glaucoma	20(24%)	13(16%)	13(15%)	9(10%)	55(16%)	
Diabetic retinopathy	10(13%)	16(20%)	19(21%)	21(23%)	66(20%)	
Macular degeneration	8(10%)	10(13%)	12(14%)	8(9%)	38(11%)	
Others	10(13%)	11(14%)	17(20%)	14(16%)	52(16%)	
**Duration of Illness**						
< 1 year	43(54%)	46(58%)	39(46%)	51(57%)	179(54%)	0.879
1–5 years	29(36%)	27(34%)	34(40%)	29(32%)	119(36%)	
5–10 years	5(6%)	4(5%)	5(6%)	7(8%)	21(6%)	
> 10 years	3(4%)	3(4%)	6(7%)	3(3%)	15(4%)	
**Other Chronic Disease**						
None	31(39%)	19(24%)	41(49%)	30(33%)	121(36%)	0.279
Diabetes	37(46%)	46(58%)	25(30%)	39(43%)	147(44%)	
Hypertension	25(31%)	34(42%)	24(29%)	43(48%)	126(38%)	
Others	7(9%)	5(6%)	2(2%)	4(4%)	18(5%)	

χ^2^ test was used for comparison between groups, α = 0.05

### Preference analysis of health management services in older adults with VI

#### Reliability analysis

Pearson's R and Kendall's Tau can be used to evaluate the gap between actual preference and predicted preference, and evaluate the success of the model test. In this study, Pearson's R statistic was greater than 0.8 and Kendall's Tau statistic was greater than 0.6. The significance level of the two-tail test was *P* < 0.05, which was statistically significant and reliable (Table 4).

**Table 4:** Preference model test results

Correlation	Total		Grade 1		Grade 2		Grade 3		Grade 4	
	Value	Sig	Value	Sig	Value	Sig	Value	Sig	Value	Sig
Pearson’s R	0.949	0.000	0.907	0.000	0.897	0.000	0.867	0.000	0.914	0.000
Kendall’s tau	0.752	0.000	0.708	0.000	0.695	0.000	0.673	0.000	0.691	0.000

Pearson’s R and Kendall’s tau: give the correlation coefficient between the score estimated from the model and the measured preference score. α = 0.05

#### Preference of attributes and levels of health management strategies in older adults with VI

**Fig. 2:**
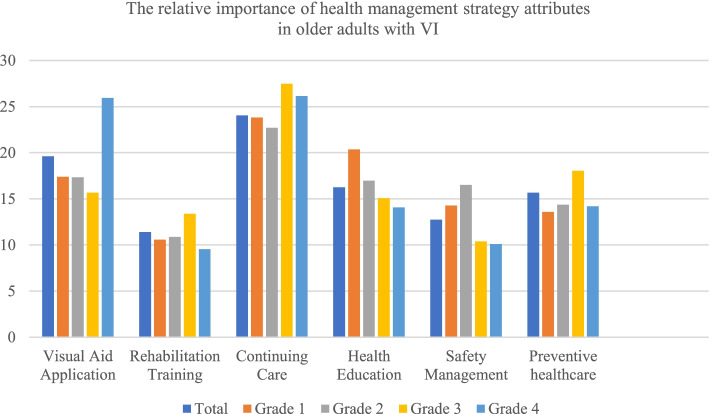
The relative importance of health management strategy attributes in older adults with VI

The relative importance of attributes of health management strategies by older adults with VI was ranked. The order was as follows: continuing care (24.033%), visual aid application (19.61%), health education (16.241%), preventive healthcare (15.667%), safety management (12.757%) and rehabilitation training (11.392%). Patients with VI of different grades ranked each attribute differently. Older adults with Grade 1 and Grade 2 VI gave more importance to safety management than older adults with Grade 3 and Grade 4 VI, accounting for 14.276% and 16.509%, respectively. Older adults with Grade 3 and Grade 4 VI give more importance to preventive healthcare than older adults with Grade 1 and Grade 2 VI, accounting for 18.035% and 14.178%, respectively (Fig. 2).

**Fig. 3:**
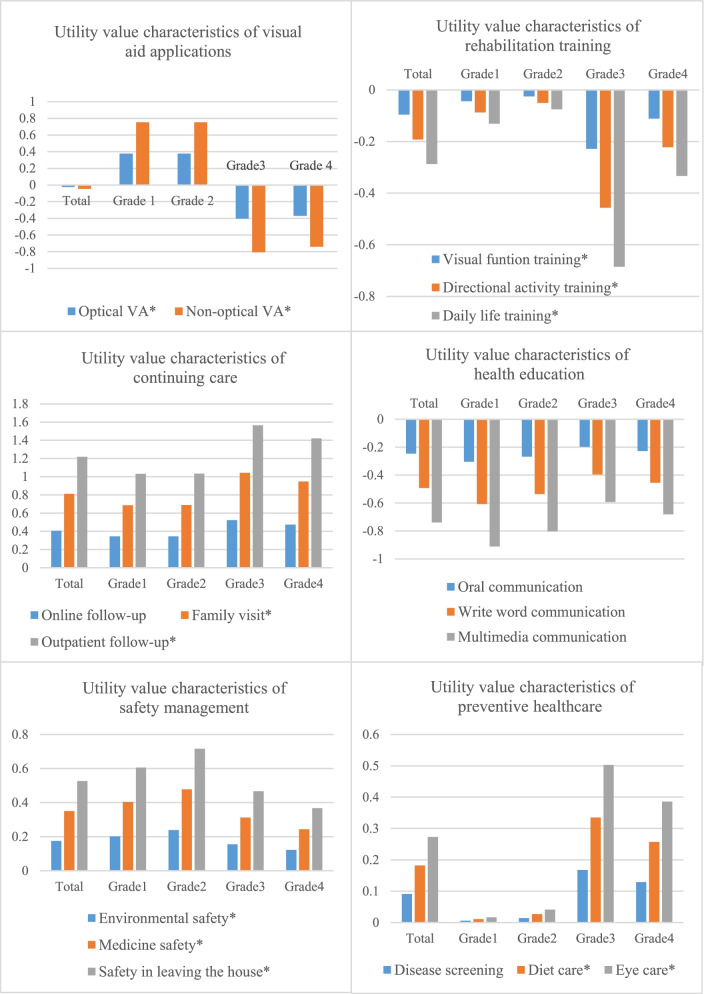
Utility values of each attribute of health management strategy in older adults with VI

Overall, the utility values of all levels in continuing care, safety management and preventive healthcare are positive, while the utility values of all levels in visual aid application, health education and rehabilitation training are negative. From the perspective of older adults with VI of different grades, the preference degree of the two levels of visual aid application was statistically significant (*P* < 0.001). Older adults with Grade 1 and Grade 2 VI preferred the visual aid application and especially non-optical visual aid, while older adults with Grade 3 and Grade 4 VI did not show a preference for the visual aid application. The utility values associated with the four grades in the three levels of rehabilitation training were all negative, with the least liked attribute being daily life training, followed by directional activity training, visual function training. Differences between grades were statistically significant (*P* < 0.001), with rehabilitation training being the least liked attribute in group of older adults with Grade 3 VI. The utility values of the three levels in continuing care were all positive, with outpatient follow-up being the most preferred attribute in all groups, followed by family visit and online follow-up. There were statistically significant differences with regards to preference of outpatient follow-up and home visit between the different groups (*P* < 0.001). The older adults with Grade 3 VI had the highest degree of preference of this attribute, although there was no statistically significant difference between groups with regards of preference for online follow-up (*P* > 0.05). The utility values of the three levels in health education were all negative, with the least liked attribute being multimedia communication, followed by written word communication and oral communication, with no statistically significant differences among the four grades (*P* > 0.05). The utility values of the three levels of safety management were all positive, with patients indicating that they preferred safety to leave the house the most, followed by medicine safety management and environmental safety management, with statistically significant differences between grades (*P* < 0.05). The utility values of three levels in preventive healthcare were positive, including eye care, diet care, and disease screening, with the differences in preference for eye care and diet care being statistically significant between grades (*P* < 0.05) and older adults with Grade 3 and Grade 4 VI indicating higher preference. Difference in preferences with relation to in disease screening were not statistically significant between grades (*P* > 0.05) (Fig. 3).

## Discussion

### Demand preferences for health management services of older adults with VI in total

Older adults with VI have the highest preference for continuing care, which ranked first in terms of relative importance among the four grades. Outpatient follow-up was the most popular attribute, a finding that is consistent with the study by Draper and colleagues [33]. This preference may be related to the professional and non-interfering working environment in outpatient clinics, which can give patients a higher sense of participation in the medical treatment. Family visit and online follow-up were also favored attributes, although to lesser degree than outpatient follow-up. The reason for this might be related to the fact that most older adults are still influenced by traditional ideas and have a higher acceptance of hospital medical treatments but a lack of trust in family visits and online follow-up. In the future, conveying the advantages of online follow-up in older adults with VI to help gain their trust is key in the development of continuing care services.

Rehabilitation training was the least favorite service indicated by older adults with VI, a result consistent with the study from Lu and colleagues [34], suggesting a lower demand for functional training in this population, which may be related to the long time spent without significant effect in the short term. Some older adults think that rehabilitation training is useless and it is not necessary. However, Zheng [35] reported that patients have a higher demand for rehabilitation training of visual function, potentially related to the type of rehabilitation training and individual characteristics of patients. Clinical practice suggests that [36–39] rehabilitation training can improve the ability to utilize residual vision, and additional forms of rehabilitation training could be explored in the future to provide targeted rehabilitation training based on patients' needs.

In this study, the three levels of health education were not favored by older adults with VI, a finding that may be related to the health literacy of the patients. Kim [40] found that the older adults are likely to be unwilling to accept health information due to their lack of interest in health management and reluctance to adjust and adapt to new health behaviors. At the same time, it is also possible that the functional limitations experienced by the older adults, such as short attention span, cognitive deficits, hinder effective health education and lead to patient frustration, discouragement and unwillingness to receive it [41]. How to design health education programs suitable for the older adults according to their characteristics and needs requires further research and investigation by medical staff.

### Demand preferences for health management services of older adults with VI at different grades

While older adults with Grade 1 and Grade 2 VI reported a preference for the application of visual aid, older adults with Grade 3 and Grade 4 VI did not like this attribute. This may be related to the fact that older adults with Grade 3 and Grade 4 VI can still use residual vision in daily life. Wearing of visual aid can also be uncomfortable, inconvenient to carry, and there may also be other reasons for the unwillingness to use visual aid. Gold et al. [42] have suggested that older adults with VI are reluctant to use visual aid due to their high cost and lack of training. The ability of patients to use residual vision can be enhanced by use of an appropriate visual aid based on professional optometry services and receipt of regular training to use visual aid [43]. Further research such as qualitative study or experimental study is necessary to increase the compliance in visual aid applications and fully leverage their benefits.

Safety management is more popular in older adults with Grade 1 and Grade 2 VI, which may be due to their severity of VI can lead to falls, collisions, burns and scalds, so effective care measures are urgently needed to ensure patient safety. Among safety management, safety in leaving the house was reported to have the highest demand, a result that consistent to the study from Lee et al. [44]. They have reported that older people with VI may develop avoidance behaviors due to the fear of falling and reduce opportunities to go outside, which is not conducive to their integration into society. How to ensure the safety in leaving the house of older adults with VI may be the further research direction.

The finding that demand preference for preventive healthcare services was higher among older adults with grade 3 and Grade 4 VI may be to a degree of VI that is still mostly in the disease development stage, leading to the patient realization of the importance of preventive healthcare. Among attributes of preventive healthcare, eye care services were preferred, probably because eye care is the most direct eye protective intervention. Similar to the study of Barman et al*.* [11], there is a higher dependence on eye care with age and as people experience VI. However, because older adults with grade 1 and grade 2 VI already experience severe VI, preventive healthcare at this stage may be of little significance for this population of patients.

### Strengths and limitations

The Strengths of this study is mainly the advantages of methodology. Conjoint analysis is an approach of design, data collection, and statistical analysis of research methods, that allows measuring the value that consumers place on products or services formed by combination of different attribute levels [19]. By using a mathematical analysis method, value can be assigned to each attribute level to evaluate the utility and relative importance of a product or service, and ultimately assess the consumer preference. Conjoint analysis was originally used to study consumer preferences, but it has been widely studied in recent years, such as in public policy and healthcare [45–47]. Patients are a special consumer group and have special preferences for medical services. Therefore, many scholars use the conjoint analysis method to study patients' preferences for medical services [48–50]. In this study, we used conjoint analysis to analyze the demand preferences for different health management services of a population of older adults with VI, to help clinical researchers to formulate appropriate health management strategies for this vulnerable population. The previous studies regarded older adults with VI mostly as a homogenous group, this study considered the differences in different degrees of VI [14, 51].

The health management strategies in this study only lists six kinds of health management services, each service only lists two to three types, and hence this approach is not comprehensive, which is a limitation of the conjoint analysis method. Under normal circumstances, the number of attributes in the conjoint analysis method is generally no more than six, and the number of attribute levels is two-four, with three being the most appropriate number, so all services cannot be discussed [31]. Given that all respondents of this study were from hospitals, and the duration of the illness was short (90% of respondents had a duration of illness of less than 5 years), selection bias might exist, which may have affected the representative of the research sample. Related studies involving communities, nursing homes and other multi-centers should be carried out in the future to validate our findings.

## Conclusion

The most popular health management service for older adults with VI was continuing care, and in particular outpatient follow-up. The least preferred service was rehabilitation training, with daily life training being the least popular. The preference of the older adults with different grades of VI is different, so medical workers can formulate corresponding health management strategies according to their different demand preferences, and carry out hierarchical health management. Services that they preferred should be satisfied as much as possible in the health management strategy, while the reasons for the services with lower preference can be explored and make targeted improvement to meet the demand preferences of them.

## Data Availability

The datasets used and analyzed during the current study are available from the corresponding author on reasonable request.

## References

[1] World Health Organization. Ageing and Health. 2018. Accessed 5 Feb 2018. https://www.ncl.ac.uk/who-we-are/strengths/ageing-health/

[2] Standardization Administration of China (2010). Classification and grade of disability of persons with disabilities.

[3] GBD 2019 Blindness and Vision Impairment Collaborators, Vision Loss Expert Group of the Global Burden of Disease Study (2021). Causes of blindness and vision impairment in 2020 and trends over 30 years, and prevalence of avoidable blindness in relation to vision 2020: The right to sight: an analysis for the global burden of disease study. Lancet Glob Health.

[4] Bourne RRA, Flaxman SR, Braithwaite T (2017). Magnitude, temporal trends, and projections of the global prevalence of blindness and distance and near vision impairment: a systematic review and meta-analysis. Lancet Glob Health.

[5] Xu T, Wang B, Liu H (2020). Prevalence and causes of vision loss in china from 1990 to 2019: Findings from the global burden of disease study 2019. Lancet Public Health.

[6] Assi L, Varadaraj V, Shakarchi AF (2020). Association of vision impairment with preventive care use among older adults in the united states. Jama Ophthalmol.

[7] Swenor BK, Lee MJ, Varadaraj V, Whitson HE, Ramulu PY (2020). Aging with vision loss: A framework for assessing the impact of visual impairment on older adults. Gerontologist.

[8] Guymer C, Casson R, Howell C, Stocks N (2017). The aged study. Age-related eye disease (aged) in south australian general practice: are we blind to early detection and intervention?. Aust J Prim Health..

[9] Sabanayagam C, Fenwick E, Ong PG (2016). Visual impairment in old and very old community-dwelling asian adults. Ophthalmol.

[10] Demmin DL, Silverstein SM (2020). Visual impairment and mental health: Unmet needs and treatment options. Clin Ophthalmol.

[11] Barman D, Mishra M (2020). How does eye care seeking behaviour change with increasing age and visual impairment? Intersectional analysis of older adults in the indian sundarbans. BMC Geriatr.

[12] Ehrlich JR, Ojeda LV, Wicker D (2017). Head-Mounted Display Technology for Low-Vision Rehabilitation and Vision Enhancement [J]. Am J Ophthalmol.

[13] Joshi MR, Persad V, Farnon N (2021). A retrospective study of causes of visual impairment and use of low vision devices in the low vision clinic in Trinidad and Tobago [J]. J Optom.

[14] Kaldenberg J, Smallfield S (2020). Occupational therapy practice guidelines for older adults with low vision [J]. Am J Occup Ther.

[15] Trauzettel-Klosinski S (2011). Current methods of visual rehabilitation [J]. Dtsch Arztebl Int.

[16] Blaylock SE, Vogtle LK (2017). Falls prevention interventions for older adults with low vision: a scoping review: Étude de portée sur les interventions visant à prévenir les chutes chez les aînés ayant une basse vision [J]. Can J Occup Ther.

[17] Jensen H, TUBæK G. Elderly people need an eye examination before entering nursing homes [J]. Dan Med J, 2017;64(2):A5325. 28157061

[18] Luu W, Kalloniatis M, Bartley E (2020). A holistic model of low vision care for improving vision-related quality of life [J]. Clin Exp Optom.

[19] Qutishat Y, Shublaq S, Masoud M (2020). Low Vision Profile in Jordan: A Vision Rehabilitation Center-Based Study [J]. Healthcare (Basel).

[20] Zeng SH. Effects of home nursing intervention on social support, self-efficacy and quality of life of patients with low vision [D]. Southern Medical University, 2016.

[21] Hu HS, Yu XR, Chen Y (2016). Research on community management model of cataract patients based on two-way management [J ]. Hospital Management Forum.

[22] Matti AI, Pesudovs K, Daly A (2011). Access to low-vision rehabilitation services: barriers and enablers [J]. Clin Exp Optom.

[23] Tan AC, Man R, Wong CW (2018). Randomized controlled trial evaluating a novel community eye care programme for elderly individuals with visual impairment [J]. Clin Exp Ophthalmol.

[24] Word Health Organization (2019). World report on vision.

[25] Ong SR, Crowston JG, Loprinzi PD, Ramulu PY (2018). Physical activity, visual impairment, and eye disease. Eye (Lond).

[26] Schakel W, Bode C, van de Ven PM, van der Aa HPA, Hulshof CTJ, van Rens G (2019). Understanding fatigue in adults with visual impairment: A path analysis study of sociodemographic, psychological and health-related factors. PLoS One..

[27] Cai MC, Zhao F, Shen D, Lyu YB, Zhang XR, Zhou JH (2020). Influence of visual impairment on mortality in the elderly aged 65 years and older in 8 longevity areas in china. Zhonghua Liu Xing Bing Xue Za Zhi.

[28] Meng X, Zhou W, Sun Z, Han Q, Zhang J, Zhang H (2021). Prevalence and causes of bilateral visual impairment in rural areas of tianjin, china - the tianjin eye study. Acta Ophthalmol.

[29] Larsen PP, Thiele S, Krohne TU, Ziemssen F, Krummenauer F, Holz FG (2019). Visual impairment and blindness in institutionalized elderly in germany. Graefes Arch Clin Exp Ophthalmol.

[30] Bin Yameen TA, Abadeh A, Slomovic J, Lichter M (2020). Visual impairment and unmet eye care needs among a syrian adult refugee population in a canadian city. Can J Ophthalmol.

[31] McCoullough D (2002). A user's guide to conjoint analysis.

[32] Sampalean NI, de-Magistris T, Rama D (2020). Investigating italian consumer preferences for different characteristics of provolone valpadana using the conjoint analysis approach. Foods.

[33] Draper EM, Feng R, Appel SD, Graboyes M, Engle E, Ciner EB (2016). Low vision rehabilitation for adult african americans in two settings. Optom Vis Sci.

[34] Lu XL, Li X, Qiu ZY, Chen D, Cheng ZW, Chen JN (2020). Unmet needs and services of rehabilitation for people with visual disability using logistic regression analysis. Chinese J Rehabil Theory Pract.

[35] Zheng HL, Lu XL, Zheng XY, Cheng MJ, Jiang WJ, Xu GX (2018). Quality of life and nursing service need in eldly low vision patients. Int Eye Sci.

[36] Bittner AK, Yoshinaga PD, Wykstra SL, Li T (2020). Telerehabilitation for people with low vision. Cochrane Database Syst Rev.

[37] Chotikavanich S, Chanvarapha N, Loket S, Yingyong R, Dongngam S, Nujoi W (2018). A 5-year retrospective record review of hospital-based low-vision rehabilitation in thailand. Clin Optom (Auckl).

[38] Laby DM (2018). Case report: Use of sports and performance vision training to benefit a low vision patient's function. Optometry Vision Sci.

[39] Sahli E, Altinbay D, Kiziltunc PB, Idil A (2021). Effectiveness of low vision rehabilitation using microperimetric acoustic biofeedback training in patients with central scotoma. Curr Eye Res.

[40] Kim MY, Oh S (2020). Nurses' perspectives on health education and health literacy of older patients. Int J Env Res Pub He.

[41] Shang X, Zhu Z, Wang W, Ha J, He M (2021). The association between vision impairment and incidence of dementia and cognitive impairment: A systematic review and meta-analysis. Ophthalmol.

[42] Gold D, Zuvela B, Hodge WG (2006). Perspectives on low vision service in canada: A pilot study. Can J Ophthalmol.

[43] Shah P, Schwartz SG, Gartner S, Scott IU, Flynn HW (2018). Low vision services: A practical guide for the clinician. Ther Adv Ophthalmol.

[44] Lee SP, Hsu YW, Andrew L, Davis T, Johnson C. Fear of falling avoidance behavior affects the inter-relationship between vision impairment and diminished mobility in community-dwelling older adults. Physiother Theor Pr. 2020:1–9. https://pubmed.ncbi.nlm.nih.gov/32543314/. 10.1080/09593985.2020.1780656 PMC8212668 32543314

[45] Anderson MM, Garman AN, Johnson TJ (2020). Understanding Student Preferences in the Selection of a Graduate Allied Health Program: A Conjoint Analysis Study [J]. J Allied Health.

[46] Larsen A, Tele A, Kumar M (2021). Mental health service preferences of patients and providers: a scoping review of conjoint analysis and discrete choice experiments from global public health literature over the last 20 years (1999–2019) [J]. BMC Health Serv Res.

[47] VerDonck N, Vander Stichele G, Huys I (2018). Learnings from consumer research for patient preference research [J]. Value Health.

[48] Diks ME, Hiligsmann M, van der Putten IM (2021). Vaccine preferences driving vaccine-decision making of different target groups: a systematic review of choice-based experiments [J]. BMC Infect Dis.

[49] Hofheinz R, Clouth J, Borchardt-Wagner J (2016). Patient preferences for palliative treatment of locally advanced or metastatic gastric cancer and adenocarcinoma of the gastroesophageal junction: A choice-based conjoint analysis study from Germany [J]. BMC Cancer.

[50] Wildenbos GA, Horenberg F, Jaspers M (2018). How do patients value and prioritize patient portal functionalities and usage factors? A conjoint analysis study with chronically ill patients [J]. BMC Med Inform Decis Mak.

[51] Larsen PP, Thiele S, Krohne TU (2019). Visual impairment and blindness in institutionalized elderly in Germany. Graefes Arch Clin Exp Ophthalmol.

